# Phase Ib study of NGR–hTNF, a selective vascular targeting agent, administered at low doses in combination with doxorubicin to patients with advanced solid tumours

**DOI:** 10.1038/sj.bjc.6605162

**Published:** 2009-06-30

**Authors:** V Gregorc, A Santoro, E Bennicelli, C J A Punt, G Citterio, J N H Timmer-Bonte, F Caligaris Cappio, A Lambiase, C Bordignon, C M L van Herpen

**Affiliations:** 1 Department of Oncology, Istituto Scientifico San Raffaele, Via Olgettina, 60, Milan 20132, Italy; 2 Department of Oncology, Istituto Clinico Humanitas, Via Manzoni, 56, Rozzano 20089, Italy; 3 Department of Oncology, Ospedale San Martino, Largo R Benzi, 10, Genoa 16132, Italy; 4 Department of Medical Oncology (452), Radboud University Nijmegen Medical Centre, PO Box 9101, HB Nijmegen 6500, The Netherlands; 5 MolMed, Via Olgettina, 58, Milan 20132, Italy

**Keywords:** NGR-hTNF, vascular targeting agent, doxorubicin

## Abstract

**Background:**

Asparagine–glycine–arginine–human tumour necrosis factor (NGR–hTNF) is a vascular targeting agent exploiting a tumour-homing peptide (NGR) that selectively binds to aminopeptidase N/CD13, overexpressed on tumour blood vessels. Significant preclinical synergy was shown between low doses of NGR-TNF and doxorubicin.

**Methods:**

The primary aim of this phase I trial was to verify the safety of low-dose NGR–hTNF combined with doxorubicin in treating refractory/resistant solid tumours. Secondary objectives included pharmacokinetics (PKs), pharmacodynamics, and clinical activity. In all 15 patients received NGR–hTNF (0.2–0.4–0.8–1.6 *μ*g m^−2^) and doxorubicin (60–75 mg m^−2^), both given intravenously every 3 weeks.

**Results:**

No dose-limiting toxicity occurred and the combination was well tolerated. Around two cases of neutropenic fevers, lasting 2 days, and two cases of cardiac ejection-fraction drops, one asymptomatic and the other symptomatic, were registered. Only 11% of the adverse events were related to NGR–hTNF and were short-lasting and mild-to-moderate in severity. There was no apparent PK interaction and the shedding of soluble TNF-receptors did not increase to 0.8 *μ*g m^−2^. One partial response (7%), at dose level 0.8 *μ*g m^−2^, and 10 stable diseases (66%), lasting for a median duration of 5.6 months, were observed.

**Conclusions:**

NGR–hTNF plus doxorubicin was administered safely and showed promising activity in patients pre-treated with anthracyclines. The dose level of 0.8 *μ*g m^−2^ NGR–hTNF plus doxorubicin 75 mg m^−2^ was selected for phase II development.

Tumour necrosis factor-alpha (TNF-*α*) was originally identified for its ability to induce apoptosis of tumour-associated endothelial cells and massive haemorrhagic necrosis of transplanted solid tumours (Carswell *et al*, 1975). Subsequent studies have shown that TNF-*α* can rapidly alter endothelial permeability and markedly decrease interstitial fluid pressure (Brett *et al*, 1989), both of which are believed to be critical for drug uptake in tumours. Disappointingly, early-stage clinical development using recombinant human TNF-*α* (hTNF-*α*) was hampered by severe systemic toxicity, the maximum tolerated dose being 10–50 times lower than the estimated effective dose (Creaven *et al*, 1989; Gamm *et al*, 1991).

To exploit a ligand-direct approach, asparagine–glycine–arginine–human tumour necrosis factor (NGR–hTNF) has been genetically engineered by coupling the N-terminus of hTNF-*α* with the C-terminus of a tumour-homing NGR-peptide, which is a ligand of the aminopeptidase N (CD13) overexpressed by endothelial cells of newly formed human tumour blood vessels (Curnis *et al*, 2000; Corti and Ponzoni, 2004). CD13 is a membrane-bound metalloprotease, and is thought to have an important role in chemokine processing and tumour invasion, and therefore is considered an attractive target for inhibiting angiogenesis. Recently, in a CD13-null mice model it was shown that although aminopeptidase-N activity is not essential for embryonic and foetal development, including *de novo* blood vessel formation (i.e., vasculogenesis) and normal adult function, it is crucial for the pathological development of new blood vessels from existing blood vessels (i.e., angiogenesis) in disease (Rangel *et al*, 2007).

In preclinical models, murine NGR–TNF showed a biphasic dose–response curve with significant anti-tumour activity even at very low doses in the nanogram range (0.005 *μ*g kg^−1^) (Curnis *et al*, 2002), equivalent in humans to a dose of 0.2 *μ*g m^−2^, which is the selected starting dose for phase I clinical development. Furthermore, it was shown that one mechanism underlying the synergism displayed by the combination between minute doses of murine NGR–TNF and doxorubicin was related to vascular barrier alteration and thus increased the uptake of this chemotherapeutic agent in tumours (Curnis *et al*, 2002). It is to be noted that NGR–TNF increased both the percentage of tumour cells that could be reached by doxorubicin in 2 h and the intracellular amount of the drug, suggesting that NGR–TNF can alter drug-penetration barriers. Moreover, the sequence of drugs and the timing of administrations are crucial for these effects. In fact, the maximal synergism was observed within a 2-h interval between the administration of NGR–TNF and subsequent chemotherapy dosing in all models and with all chemotherapeutics tested (Sacchi *et al*, 2006).

Recently, the phase I dose-escalation study, exploring 17 dose levels ranging from 0.2 to 60 *μ*g m^−2^ in 70 patients, established the maximum tolerated dose (MTD) of NGR–hTNF at 45 *μ*g m^−2^, when given as a single agent once every 3 weeks, with dose-limiting toxicities at 60 *μ*g m^−2^ being characterised by grade 3 dyspnoea and acute infusion reaction (van Laarhoven *et al*, 2008).

In light of the promising preclinical findings, the present phase I clinical trial was designed to determine a safe low dose of NGR–hTNF in combination with the standard dose of doxorubicin.

## Patients and methods

### Eligibility criteria

The following inclusion criteria had to be met: age ⩾18 years; histologically or cytologically confirmed solid tumour not amenable to any clinical improvement by current standard treatments; Eastern Cooperative Oncology Group (ECOG) performance status ⩽1; normal bone marrow, hepatic, and renal function; previous treatment with a lifetime cumulative dose of anthracyclines <300 mg m^−2^ (to allow the administration of an adequate number of cycles). Patients were excluded if they had significant cardiovascular disease, including a baseline cardiac ejection fraction (EF) <55%, or known central nervous system (CNS) metastases, as were patients who completed radiotherapy or systemic therapy within 4 weeks or underwent surgery within 2 weeks of the start of NGR–hTNF treatment. All patients provided written informed consent, and the protocol was approved by institutional ethics review committees.

### Study design and dosing

This was a multicentre, multinational, phase I dose-escalation study with three patients who were administered each of four sequentially increasing low-dose levels of NGR–hTNF (0.2–0.4–0.8–1.6 *μ*g m^−2^), given intravenously through a 1-h infusion every 3 weeks, in combination with a fixed dose of doxorubicin (75 mg m^−2^), given through a 15-min intravenous infusion, 2 h after the start of NGR–hTNF infusion. In the first cohort, NGR–hTNF was given at the starting dose of 0.2 mg m^−2^ in combination with a lower dose (−20%) of doxorubicin (60 mg m^−2^). The maximum cumulative dose of doxorubicin was capped at 550 mg m^−2^. Patients with continued clinical response or stable disease were eligible to receive NGR–hTNF as a single agent until progressive disease or unacceptable toxicity.

The primary objective was to verify the safety of low doses of NGR–hTNF in combination with doxorubicin for the establishment of presence or absence of dose-limiting toxicities (DLTs). Secondary aims included a pharmacokinetic (PK) analysis of NGR–hTNF and doxorubicin, monitoring of soluble TNF receptor (sTNF-RI and sTNF-RII) levels, and preliminary anti-tumour activity assessment.

### Dose-limiting toxicities

Dose-limiting toxicities applicable to the study were defined as adverse events occurring during the first cycle and fulfilling one of the following criteria: any grade 3–4 non-haematological toxicity, with the exclusion of alopecia, nausea, vomiting, and fever, which could be rapidly controlled with appropriate measures; an absolute neutrophil count (ANC) <500 *μ*l^−1^ lasting for ⩾7 days; a neutropenic fever defined as ANC<1000 *μ*l^−1^ and fever>38.5°C; a thrombocytopenia ⩽25 000/*μ*l or thrombocytopenic bleeding requiring transfusion; and a severe hypotension requiring dopamine administration.

A standard 3+3 escalation design was followed. At each dose level, the first patient had to complete one cycle before subsequent patients could be included. To increase the dose to the next dose level, the three patients enrolled at the previous dose level had to complete the first cycle. To fully document the safety profile, an expansion of the cohorts to six patients was planned the presence of one DLT. Intolerable dosage was defined as two or more out of six patients experiencing DLT and then the immediate lower dose level was considered as the maximum tolerated dose.

### Pharmacokinetics and pharmacodynamics

Pharmacokinetic blood sampling was carried out on day 1 with samples drawn before infusion and at 15, 30, 60, 90, and 120 (just before doxorubicin administration), 134 (1 min before the end of doxorubicin administration), 180, 240, and 360 min after the first three cycles. NGR–hTNF and soluble plasma TNF receptors (sTNF-RI and sTNF-RII) were computed by using an enzyme-linked immunosorbent assay and values obtained at different time points after each cycle were normalised by subtracting the time-zero value. The bioanalytical assay of doxorubicin and its metabolite doxorubicinol was carried out using liquid chromatography–tandem mass spectrometry. Maximum plasma concentration (*C*_max_) and area under the plasma concentration–time curve to the last detectable concentration (AUC_0−*t* last_) were estimated from plasma concentration–time data using the standard non-compartmental methods.

### Tumour assessment and safety

Tumour measurements were carried out at baseline and every 6 weeks until progressive disease. Measurable target lesions were evaluated for response using Response Evaluation Criteria in Solid Tumours (RECIST), and the duration of stable disease was measured from the start of therapy until the criteria for progression were met. Adverse events were recorded from day 1 till 28 days after the last dose, and were graded on the basis of the Common Terminology Criteria for Adverse Events (CTCAEs), version 3.0. Complete blood counts were assessed weekly while patients were on therapy. Assessment of left ventricular ejection fraction (LVEF) by echocardiogram was carried out before treatment and every other cycle. Doxorubicin was planned to be discontinued in patients who had experienced either a fall in LVEF from baseline ⩾20% or a fall to ⩽45%.

## Results

### Patients

A total of 15 patients (5 women and 10 men), with a median age of 58 years (range, 31–82 years) and a performance status of 0 (60%) or 1 (40%), were enrolled between March 2006 and November 2006. All patients were heavily pre-treated: all had earlier received chemotherapy (median of two regimens; range, 1–6), including 60% (*n*=9) of the patients previously treated with an anthracycline-containing regimen, 86% before surgery, and 40% before radiotherapy.

### Safety and tolerability

In total, 89 cycles of NGR–hTNF (median, 6; range, 2–15) and 62 cycles of doxorubicin (median, 4; range, 2–8) were administered. According to the study protocol, six patients discontinued doxorubicin when they reached the maximum cumulative dose (i.e., 550 mg m^−2^). All patients received at least one dose of study drugs and were assessable for toxicity.

No DLTs occurred and, as expected for this low-dose range of NGR–hTNF, MTD was not reached. Globally, treatment discontinuations were a result of progressive disease in 14 patients (93%) and an adverse event (pulmonary embolism) in 1 patient (7%).

**Figure 1:**
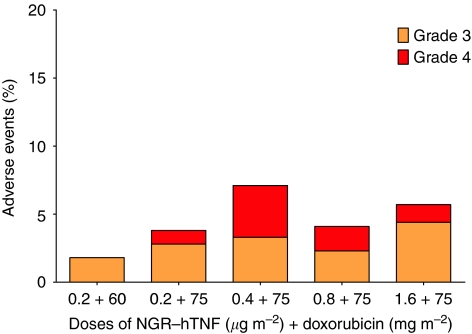
Incidence of grade 3–4 adverse events by dose levels. For each dose level of asparagine–glycine–arginine–human tumour necrosis factor (NGR-hTNF) and doxorubicin the following number of cycles were administered, respectively: 0.2–60 : 12–12; 0.2–75 : 27–17; 0.4–75 : 13–11; 0.8–75 : 25–12; 1.6–75 : 12–10.

The majority of haematological (29%) and non-haematological (71%) adverse events were mild (49%) to moderate (28%) in severity, with grade 3 and 4 being observed in 15% and 8%, respectively. The adverse events occurring in ⩾20% of patients are listed in Table 1 and the incidence of grade 3–4 toxicities by dose levels and number of cycles is depicted in Figure 1.

The most commonly reported non-haematological adverse events were nausea, asthenia, pain, and vomiting, and these were mainly considered to be related to doxorubicin administration.

Haematological toxicity was assessed weekly throughout the study. In all eight patients (all treated with doxorubicin 75 mg m^−2^) experienced grade 4 neutropenia in 17 cycles. However, neutropenia was generally short-lasting and reversible. There were only two neutropenic fever episodes, both occurring 2 weeks after the third cycle, lasting 2 days and recovering without sequelae. Three patients required haematopoietic growth factor support for neutropenia. A 20% dose reduction of doxorubicin was required in four patients for 10 cycles and a dose delay was registered in four patients for 7 cycles. No grade 4 anaemia or thrombocytopenia occurred.

**Figure 2:**
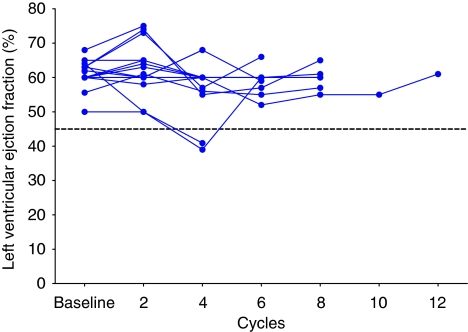
Left ventricular ejection fraction (LVEF) values over time for all patients.

All patients underwent LVEF assessment by echocardiogram every other cycle and the LVEF profile over time for all patients is illustrated in Figure 2. The EF values changed in only 2 of 15 patients assessed. An asymptomatic drop in LVEF from 50% to 40% was observed in an 83-year-old patient after the fourth cycle (lifetime cumulative doxorubicin dose of 500 mg m^−2^), with a subsequent recovery to 60% recorded after two additional courses of NGR–hTNF monotherapy and following doxorubicin discontinuation. This patient, who was previously treated with liposomal doxorubicin, also experienced a grade 3 febrile neutropenia after the third cycle and was withdrawn from the study after the sixth cycle owing to progressive disease. An additional symptomatic drop in LVEF from 50% to 40% was registered in a 62-year-old male patient with small-cell lung cancer and with a positive history of hypertension and aortic aneurysm. He had been previously treated with doxorubicin. After the fourth cycle (lifetime cumulative doxorubicin dose of 400 mg m^−2^), he had progressive disease and was hospitalised with a diagnosis of acute myocardial ischaemia. A coronarography revealed a thrombotic occlusion of the left coronary artery treated with angioplasty and stent placement. He was discharged after 3 weeks following an improvement of his clinical conditions. In both patients, neither anaemia nor other non-haematological toxicities of grade 3–4 were detected. Only 42 (11%) out of 390 adverse events were considered to be related to NGR–hTNF. All of these events were of mild (67%) to moderate (33%) intensity. About seven patients (grade 1, *n*=3; grade 2, *n*=4) experienced rigors/chills in 15 cycles, and three patients (grade 1, *n*=2; grade 2, *n*=1) experienced a transient blood pressure increase in 3 cycles. These events seemed to be dose unrelated to, short-lasting, and presented a clear temporal relationship between their onset and drug infusion, as they generally occurred about 30–40 min after the start of infusion and lasted for approximately 30 min.

### Serum PKs and pharmacodynamics

**Figure 3:**
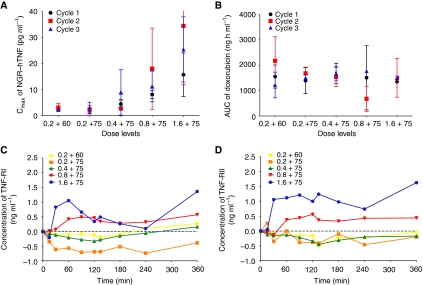
Mean asparagine–glycine–arginine–human tumour necrosis factor (NGR-hTNF) *C*_max_ (**A**) and doxorubicin AUC (**B**) during the first three cycles by dose level. Mean plasma concentrations of soluble TNF receptors tumour necrosis factor receptor I (TNF-RI) (**C**) and tumour necrosis factor receptor II (TNF-RII) (**D**) during the first cycle by dose level. Abbreviations: *C*_max_, maximal plasma concentration; AUC, area under the plasma concentration *vs* time curve up to the last detectable concentration.

The mean *C*_max_ values of NGR–hTNF and AUC_0−*t* last_ of doxorubicin during the first three cycles for each dose level are shown in Figures 3A and B. The average systemic exposure to NGR–hTNF during cycles 2 and 3 was comparable to that found during the first cycle and increased in a dose- and exposure-proportionate manner. The mean (±s.d.) NGR–hTNF *C*_max_ achieved following the first cycle at doses of 0.2 (plus doxorubicin 60 mg m^−2^), 0.2 (plus doxorubicin 75 mg m^−2^), 0.4, 0.8, and 1.6 *μ*g m^−2^ was 2.22 (±0.58), 1.82 (±0.90), 4.44 (±1.70), 8.10 (±1.70), and 15.70 (±1.34) pg ml^−1^, respectively.

The apparent terminal half-life (*t*_1/2_) of NGR–hTNF was relatively short, with means±s.d. ranging from 0.99±0.83 h (dose level 0.2 *μ*g m^−2^) to 2.56±0.65 h (dose level 0.8 *μ*g m^−2^). With the exception of slightly more prolonged *t*_1/2_ values reported in the present combination study, NGR–hTNF Pks were in reasonable agreement with those found in a low-dose, single-agent phase I study (Gallo-Stampino *et al*, 2007).

Consistently, doxorubicin systemic exposure was comparable among the five cohorts of patients who received the dose of 60 or 75 mg m^−2^, irrespective of the NGR–hTNF dose given in combination, and between cycles 2 and 3 *vs* cycle 1. Overall, no apparent changes in the Pks of NGR–hTNF and doxorubicin were detected by administering the two drugs in combination.

During the first cycle of treatment at 0.2 and 0.4 *μ*g m^−2^, both the maximum (*E*_max_) and the average (*E*_av_) stimulatory effects attributed to NGR–hTNF on the plasma levels of sTNF-RI were scattered around 0, indicating neither stimulation nor inhibition effects of NGR–hTNF on the concentrations of the receptors. The levels of the receptor were slightly above 0 at 0.8 *μ*g m^−2^ dose, whereas an increase was observed only at 1.6 mg m^−2^. For sTNF-RII a similar behaviour was noted. However, at the highest dose level tested (1.6 mg m^−2^), the stimulation of the concentrations of sTNF-RII by NGR–hTNF was about two times higher than that of sTNF-RI (Figures 3C and D). Moreover, at this dose level *E*_max_ for both receptors was achieved around the time of attainment of NGR–hTNF maximal plasma concentration. No circulating antibodies to NGR–hTNF were detected.

### Anti-tumour activity

**Figure 4:**
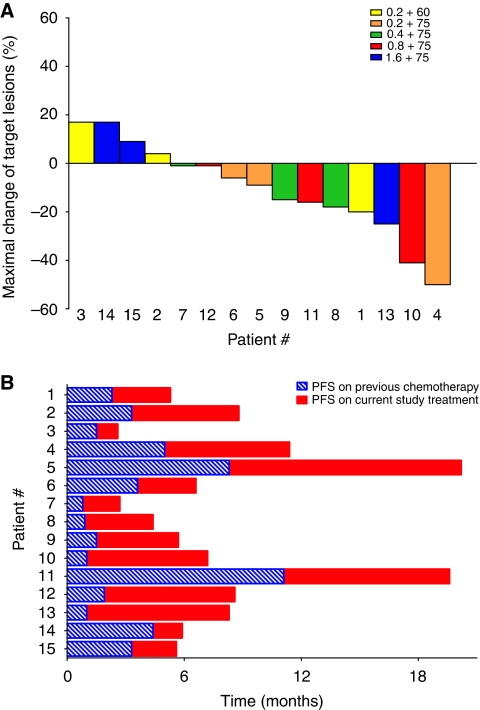
Waterfall diagram showing maximal changes of target lesions by dose levels (**A**) and progression-free survival (PFS) durations while on the previous regimen and on the current study treatment (**B**).

One partial response was documented at the fourth dose level (NGR–hTNF 0.8 *μ*g m^−2^ + doxorubicin 75 mg m^−2^) in a patient with oesophageal cancer and lung metastases, who was previously treated with two cisplatin- and fluorouracil-based regimens. An unconfirmed partial response was registered after the sixth cycle in an ovarian cancer patient who had previously received five chemotherapy regimens. Stable disease as the best response was reported in further 9 patients with a median duration of 5.6 months (Table 2). Off these patients with stable disease, the maximum changes in their target lesions ranged from 40% shrinkage to 9% growth (Figure 4A). Overall, the disease control rate (partial response + stable disease) in these patients who were resistant to standard chemotherapy, including 9 patients who were previously treated with an anthracycline-based regimen, was 73% (11 out of 15 patients). Median progression-free survival for the intent-to-treat patient population was 5.5 months, with some patients experiencing progression-free durations longer than those experienced while on the immediately previous regimen administered (Figure 4B).

## Discussion

In the present study, no dose-limiting toxicities were observed and the combination of low-dose NGR–hTNF with the standard-dose doxorubicin was safe and well tolerated, without an apparent exacerbation of the well-known toxicity profile associated to doxorubicin. In particular, NGR–hTNF did not appear to increase the frequency or severity of doxorubicin-related cardiac toxicity as measured by LVEF. Further analysis of this toxicity, however, would necessitate longer treatment durations in a larger patient population, monitoring of serial B-type natriuretic peptide and troponin levels, and the assessment of QT interval prolongation (Carver *et al*, 2007).

Moreover, only 11% of adverse events were considered to be related to NGR–hTNF, and it should be noted that these events were limited to manageable, short-lasting, and infusion time-related constitutional symptoms.

The combination of molecularly targeted agents with chemotherapy raises issues about the dose selection during early-stage clinical development. Indeed, some of the guideposts in combining chemotherapeutic agents (e.g., definition of MTD and avoidance of overlapping toxicities) might not be suitable when combined with biological agents that have low-toxicity profiles when used at the low-dose range. Moreover, given their mainly cytostatic nature, maximal anti-tumour activity could not necessarily coincide only with MTD, but also with lower drug dose, that is, the optimal biological dose (Kwak *et al*, 2007). Accordingly, the rationale of this phase I study was based on a preclinical model showing that even minute amounts of murine NGR–TNF were able to induce synergistic anti-tumour activity in combination with doxorubicin, mainly by damaging the tumour capillary network and increasing the chemotherapeutic drug uptake in the tumour (Curnis *et al*, 2002).

Although not directly measured in this study, the anti-vascular effects of NGR–hTNF have been previously observed by dynamic contrast-enhanced magnetic resonance imaging (DCE-MRI) in the dose-escalation phase I trial (van Laarhoven *et al*, 2008) and in an additional single-agent phase I trial further exploring the low-dose range (Gallo-Stampino *et al*, 2007) and, therefore, could be considered supportive of the putative mechanism of synergism shown by NGR–hTNF and doxorubicin. However, the synergism of anti-vascular agents and chemotherapy could be alternatively attributable simply to targeting two distinct cell populations (Horsman and Siemann, 2006).

Notwithstanding the fact that optimal biological dose selection is challenging, it is interesting to note that the aforementioned low-dose single-agent phase I trial selected the dose of 0.8 *μ*g m^−2^ for further development, based on a more pronounced anti-vascular effect observed at this dose and the lack of shedding of soluble TNF-receptors registered up to this dose level. These soluble receptors can compete for TNF-*α* with the cell-surface receptors, thus blocking its bioavailability and activity, with the amount and speed of receptor shedding being linearly correlated with the serum TNF-*α* levels (Aderka *et al*, 1998). Similarly, no significant shedding of circulating TNF-*α* receptors was observed up to the dose of 0.8 *μ*g m^−2^ in this study. Furthermore, patients enrolled in this dose cohort experienced a low incidence of grade 3–4 toxicity and promising disease control.

Even though anti-tumour activity was not a primary end point of this study, the high disease control rate (73%) achieved in a population heavily pre-treated with chemotherapy, including 9 patients (60%) with an anthracycline-based regimen, seem to be promising by also taking into account the minimal toxicity profile associated to NGR–hTNF and the apparent absence of overlapping toxicity with doxorubicin.

In conclusion, the present phase I trial showed that the combination of low-dose NGR-hTNF and standard-dose doxorubicin is feasible, safe, and well tolerated when administered to patients heavily pre-treated with chemotherapy, including anthracyclines. The observed safety profile and anti-tumour activity warrant further phase II clinical exploration of NGR–hTNF 0.8 *μ*g m^−2^ and doxorubicin 75 mg m^−2^ in anthracycline-sensitive solid tumours.

## Figures and Tables

**Table 1:** Haematological and non-haematological adverse events occurring in ⩾20% of patients or reaching grade 4 severity

**Adverse event**	**Any grade, *n*=15 (%)**	**Grade 1 *n* (%)**	**Grade 2 *n* (%)**	**Grade 3 *n* (%)**	**Grade 4 *n* (%)**
*Haematological adverse events*					
Neutropenia	13 (86)	—	2 (13)	3 (20)	8 (53)
Anemia	11 (73)	3 (20)	6 (40)	2 (13)	—
Leukopenia	11 (73)	—	3 (20)	5 (33)	3 (20)
Lymphopenia	5 (33)	1 (7)	1 (7)	2 (13)	1 (7)
Thrombocytopenia	2 (13)	—	1 (7)	1 (7)	—
Neutropenic fever	2 (13)	—	—	2 (13)	—
					
*Non-haematological adverse events*					
Nausea	12 (80)	10 (67)	2 (13)	—	—
Asthenia	10 (67)	6 (40)	3 (20)	1 (7)	—
Pain	8 (53)	3 (20)	3 (20)	1 (7)	1 (7)
Vomiting	8 (53)	4 (27)	3 (20)	1 (7)	—
Chills	7 (47)	3 (20)	4 (27)	—	—
Cough	5 (33)	4 (27)	—	1 (7)	—
Fever	5 (33)	4 (27)	1 (7)	—	—
Anorexia	5 (33)	3 (20)	1 (7)	1 (7)	
Alopecia	5 (33)	4 (27)	1 (7)	—	—
Constipation	4 (27)	1 (7)	3 (20)	—	—
Mucositis	4 (27)	2 (13)	2 (13)	—	—
Insomnia	4 (27)	3 (20)	1 (7)	—	—
GGTP increase	3 (20)	1 (7)	1 (7)	1 (7)	
Gastritis	3 (20)	1 (7)	2 (13)	—	—
Headache	3 (20)	1 (7)	2 (13)	—	—
Dysphagia	3 (20)	3 (20)	—	—	—
AMI	1 (7)	—	—	—	1 (7)
Pulmonary embolism	1 (7)	—	—	—	1 (7)

Abbreviations: AMI, acute myocardial ischaemia; GGTP, gamma-glutamyl transferase.

**Table 2:** Anti tumour activity by dose levels

**DL**	**Pt. no.**	**Gender/age (yrs)**	**Primary tumour**	**Previous no. of regimens**	**Previous anthracyclines/ best response**	**NGR–TNF dose (*μ*g m^−2^)/no. of cycles**	**Doxorubicin dose (mg m^−2^)/no. of cycles**	**Best response**	**Duration of PR or SD (months)**
1	1	M/58	Head and neck	2	No	0.2/4	60/4	SD	3
	2	F/42	Cervix	6	No	0.2/6	60/6	SD	4.7
	3	F/59	Colon	2	No	0.2/2	60/2	PD	—
2	4	F/58	Ovarian	5	Yes/SD	0.2/8	75/5	SD^a^	6.4
	5	M/56	Ampulla of Vater	1	No	0.2/15	75/8	SD	11.9
	6	F/31	Thymoma	1	Yes/PD	0.2/4	75/4	SD	2.9
3	7	M/58	Thymoma	1	Yes/PD	0.4/3	75/3	PD^b^	—
	8	M/62	SCLC	2	Yes/PD	0.4/4	75/4	SD	3.5
	9	M/83	Angiosarcoma	3	Yes/PD	0.4/6	75/4	SD	4.2
4	10	M/59	Oesophageal	2	No	0.8/8	75/6	PR	4.7
	11	M/54	Chordoma	5	Yes/PD	0.8/9	75/3	SD	8.2
	12	F/54	Sarcoma	2	Yes^c^	0.8/8	75/3	SD	6.5
5	13	M/51	Hepatocellular	1	Yes^d^	1.6/8	75/6	SD	6.7
	14	M/44	Melanoma	2	No	1.6/2	75/2	PD	—
	15	M/69	Sarcoma	2	Yes/PD	1.6/2	75/2	PD^b^	—

Abbreviations: DL, dose level; F, female; M, male; NGR–TNF, asparagine–glycine–arginine–tumour necrosis factor; PR, partial response; SCLC, small cell lung cancer, PD, progressive disease; SD, stable disease.

a Patient with radiologically documented partial response after the sixth cycle.

b Patient with radiologically documented stable disease at first tumour restaging carried out after two cycles.

c Patient treated with epirubicin in adjuvant setting.

d Patient with radiologically documented stable disease for whom epirubicin treatment was stopped after two cycles because of marked increase of alpha-fetoprotein.
